# Neuropsychiatric Manifestations in Thyroid and Sex Hormone Disorders: A Comprehensive Review

**DOI:** 10.1192/j.eurpsy.2025.632

**Published:** 2025-08-26

**Authors:** C. Kalogirou, C. Christou, V. Zacharopoulou, A. Potamianou, T. Soumala, G. P. Chrousos

**Affiliations:** 1 University of Patras, Patras, Greece; 2 Marien hospital, Dusseldorf, Germany; 3 Ηοspital of Leros, Leros; 4 University of Ioannina, Ioannina; 5 Medical School, University of Athens, Athens, Greece

## Abstract

**Introduction:**

Thyroid and sex hormones play pivotal roles in the regulation of various physiological processes, including brain function. Dysregulation of these hormones has been increasingly associated with a range of neuropsychiatric disorders, including depression, anxiety, cognitive impairment, and mood disorders.

**Objectives:**

This review aims to systematically examine the correlation between thyroid and sex hormones disorders and the spectrum of emerging neuropsychiatric manifestations, enlightening the pathophysiological mechanisms.

**Methods:**

A literature search was performed in many databases including PubMed, Web of Science, and Google Scholar for studies published in recent years. Eligible randomized controlled trials, observational studies, and systematic reviews examining neuropsychiatric outcomes in patients with thyroid or sex hormone disorders were included. Findings were synthesized both quantitatively, with meta-analyses where possible, and qualitatively, with thematic analysis for heterogeneous data.

**Results:**

The review identified a strong association between thyroid dysfunctions and neuropsychiatric disorders such as depression, anxiety, and cognitive decline. Hypothyroidism was consistently linked with depressive symptoms likely due to impaired serotonergic and dopaminergic neurotransmission, along with decreased hippocampal neurogenesis. Conversely, hyperthyroidism, characterized by elevated thyroid hormone levels, was associated with heightened anxiety, irritability, and emotional lability, possibly through dysregulation of the hypothalamic-pituitary-adrenal (HPA) axis and increased sympathetic nervous system activity.

In the context of sex hormone disorders, estrogen deficiency during menopause was correlated with a significant increase in behavioral and cognitive impairments, potentially mediated by reduced modulation of serotonin receptors, diminished synaptic plasticity, and increased neuroinflammatory responses. Similarly, testosterone decline in aging men was linked to mood and cognitive disorders, with evidence pointing to disruptions in androgen receptor signaling and alterations in γ-aminobutyric acid (GABA)ergic and glutamatergic pathways.

**Conclusions:**

This review underscores the significant link between thyroid dysfunctions, particularly hypothyroidism and hyperthyroidism and mood disorders such as depression and anxiety, while also indicates that estrogen deficiency and testosterone decline contribute to cognitive impairments and emotional disturbances. These findings help the healthcare providers to recognize neuropsychiatric symptoms as potential indicators of underlying endocrine disorders.

**Disclosure of Interest:**

None Declared

